# Digital participation of brain tumour patients in the assessment and treatment of communication disorders

**DOI:** 10.3389/fpsyg.2023.1287747

**Published:** 2024-01-08

**Authors:** Carolin Weiss Lucas, Sophia Kochs, Johanna Jost, Ricardo Loução, Martin Kocher, Roland Goldbrunner, Dorothee Wiewrodt, Kristina Jonas

**Affiliations:** ^1^Centre for Neurosurgery, Department of General Neurosurgery, Faculty of Medicine, University and University Hospital of Cologne, Cologne, Germany; ^2^Department of Neurosurgery, University Hospital Münster, Münster, Germany; ^3^Centre for Neurosurgery, Department of Stereotactic and Functional Neurosurgery, Faculty of Medicine, University and University Hospital of Cologne, Cologne, Germany; ^4^Department of Special Education and Rehabilitation, Faculty of Human Sciences, University of Cologne, Cologne, Germany

**Keywords:** telemedicine, remote, speech, language, neurocognitive, video conference, glioma, neuro-oncology

## Abstract

**Introduction:**

Communication deficits have a severe impact on our social interactions and health-related quality of life. Subtle communication deficits are frequently overlooked or neglected in brain tumour patients, due to insufficient diagnostics. Digital tools may represent a valuable adjunct to the conventional assessment or therapy setting but might not be readily suitable for every patient.

**Methods:**

This article summarises results of three surveys on the readiness for telemedicine among (a) patients diagnosed with high-grade glioma, (b) matched controls, and (c) speech and language therapists. The respective surveys assessed the motivation for participation in telemedical assessments and supposed influencing factors, and the use potential of digital assessment and therapy technologies in daily routine, with a spotlight on brain tumour patients and the future prospects of respective telemedical interventions. Respondents included 56 high-grade glioma patients (age median: 59 years; 48% males), 73 propensity-score matched neurologically healthy controls who were instructed to imagine themselves with a severe disease, and 23 speech and language therapists (61% <35 years; all females).

**Results and discussion:**

The vast majority of the interviewed high-grade glioma (HGG) patients was open to digitisation, felt well-equipped and sufficiently skilled. The factorial analysis showed that digital offers would be of particular interest for patients in reduced general health condition (*p* = 0.03) and those who live far from specialised treatment services (*p* = 0.03). The particular motivation of these subgroups seemed to outweigh the effects of age, equipment and internet skills, which were only significant in the control cohort. The therapists' survey demonstrated a broad consensus on the need for improving the therapy access of brain tumour patients (64%) and strengthening their respective digital participation (78%), although digitisation seems to have yet hardly entered the therapists' daily practise. In summary, the combined results of the surveys call for a joint effort to enhance the prerequisites for digital participation of patients with neurogenic communication disorders, particularly in the context of heavily burdened HGG patients with limited mobility.

## 1 Introduction

Communication is an essential part of our life and, thus, communication deficits (in the sense of impaired voice, speech, and language functions, pragmatics, and general communication skills) heavily affect health-related quality of life (Hilari and Byng, 2009; Neumann et al., 2019). Social participation can be significantly restricted depending on the severity of the communicative impairment and the underlying individual deficits, i.e., limitations in oral and written language production and reception, vocal and speech motor functions, and pragmatics (Baylor et al., 2011; Jin et al., 2021). This makes it even more difficult to maintain social relationships (Palmer et al., 2016, 2019). Unfortunately, communication disorders are common in patients with cerebral lesions like brain tumours, depending on their location and a variety of patient- and tumour-specific factors (Thomas et al., 1995; Kirkman et al., 2022; Ueda et al., 2022; Heinzel et al., 2023).

Timely detection of communication deficits using adequate diagnostic tests is crucial to identify the need for therapy and support. However, this leads to a dilemma. On the one hand, participation in (extensive) speech, language and communication examinations, and therapy sessions, can be challenging in heavily burdened and/or physically impaired patients. On the other hand, lack of participation in regular examinations results in missed diagnoses, leading to delayed or missing treatment of the respective communication deficits and associated limitations in self-sufficiency, social participation, and quality of life (Neumann et al., 2019; Palmer et al., 2019).

This is particularly relevant for brain tumour patients. This patient cohort usually suffers not only from a substantial psycho-oncological burden, even in cases of rather benign tumours (Jungk et al., 2021), but is also subjected to a demanding treatment schedule, including radiation- and chemotherapy, especially in the 1st months after tumour diagnosis and initiation of the neuro-oncological treatments. These factors might be primary reasons for the overall low rate of comprehensive neurocognitive assessments in these patients (Weiss Lucas et al., 2021).

Nowadays digitisation is progressing more and more in all areas of life. However, this entails challenges of digital participation, not only in and through digital technologies, but also within the digital world (United Nations, 2021; Jaecks and Jonas, 2022). In particular, the possibility of digital participation through respective technologies opens up new opportunities and can improve access to certain services, i.e., to the above-described diagnostic deficiency in brain tumour patients. Moreover, the use of digital technologies offers a pragmatic approach to overcoming spatial distances and organisational challenges, thereby improving access to (regular) appointments related to care. Such approaches have recently been reviewed and reported as a promising complement to traditional psychological support programs (Ownsworth et al., 2021). Likewise, they could help enhance patients' access to, e.g., speech and language therapy for patients with communication disorders. For instance, digital diagnostics could address transportation issues, especially in cases of patients with limited mobility or reduced general health conditions. Moreover, it could provide a pragmatical solution to the logistic challenge of integrating time-consuming face-to-face assessments and interventions in the hospital setting, which require a quiet room and focused participation over a relatively long period of time. Lastly, it could separate the testing or therapy setting from the often psychologically distressing hospital environment (cf. Wahl and Jankowski, 2019; Lauer, 2020).

One possible concern could be that brain tumours primarily affect elderly individuals, with a median age around 65 years and a peak incidence in the 7th decade of life (Tamimi and Juweid, 2017) —an age group which is commonly perceived to have relatively limited access and experience related to modern technologies, such as telemedicine applications and internet skills in general (Berner et al., 2020; Medienpädagogischer Forschungsverbund Südwest, 2021).

Over the recent years, a still very limited number of digital speech and language therapy tools have been reported for use in this context, beyond the pure telemedicine approach of conducting patient visits via video call, e.g., the LingoTalk app (Heide et al., 2023), the neolexon apps (Thunstedt et al., 2020), the Constant Therapy (Braley et al., 2021), and the ORLA (Cherney et al., 2021) digital/computer programs, as well as more recent virtual technology approaches (e.g., Marshall et al., 2020; Repetto et al., 2021). Further promising tools are still in development or awaiting publication, e.g., the TELL (Corsten and Iserloh, n.d.) and the Dysartrain (Klose and John, n.d.) platforms for interactive digital speech and language therapy. To date, there is little consensus regarding the equivalence/validy of such instruments, and recommendations regarding the use of distinct tools are missing.

This research project therefore deals with two main objectives:

1. We set out to investigate the openness of age-matched subjects and HGG patients to telemedical participation. We hypothesised that the overall motivation for telemedical participation might be higher in a real-life scenario (i.e., HGG patients' perspective) compared to the imaginary severe disease context (i.e., healthy subjects' perspective), especially for patients with reduced mobility, far residency-to-hospital distance, and considerable (physical or mental) disability. Moreover, we assumed that the presence of technical resources and skills, as along with factors such as young age, male gender, and high educational level, might positively influence the receptiveness towards a telemedicine setting (Medienpädagogischer Forschungsverbund Südwest, 2021).
2. We also explored the extent to which telemedicine approaches have already been incorporated into the daily routine practise of speech and language therapy professionals, both in general and particularly related to brain tumour patients with communication deficits.

## 2 Materials and methods

The research project was divided into three anonymous survey phases targeting: (I) 75 healthy controls, (II) 50 HGG patients, and (III) 20 German speech and language therapists. In the first phase, subjects were asked to imagine being severely ill and provide their estimated motivation to participate in telemedical assessments. In the second phase (real-life scenario), HGG patients were asked to estimate their motivation for such participation. Throughout both phases, important influencing factors, such as access to technical equipment, knowledge, and mobility were assessed to inform the factorial analysis. In the third phase, the use of telemedicine and other digital assessments and instruments in daily diagnostic and therapeutic practise for neurogenic communication disorders was evaluated among speech and language therapists, with a particular focus on the assessment and treatment of brain tumour patients.

### 2.1 Participants

#### 2.1.1 Healthy participants

Healthy adults (of 18 or more years of age) were recruited from an institutional database and by public calls using print and social media in January 2021. To enable a well-balanced 1:1 matching whilst optimising the ratio between study effort and useable data, we pursued a strategy of age- and gender-stratified recruitment of the healthy cohort. To construct a matched cohort corresponding to the median age of HGG patients (~65 years; Tamimi and Juweid, 2017), we planned to recruit 75 neurologically healthy respondents (i.e., 3: 2 matching of controls and HGG patients). Participants were contacted via phone call at least 24 h prior to survey execution, and were asked about their willingness to participate. Important inclusion criteria were German language as mother tongue as well as the absence of relevant neurological, communicative, and/or other neurocognitive deficits.

#### 2.1.2 Patients

HGG patients were recruited between March and August 2021 with the intention of including ~50 respondents in this study. Participants were identified using institutional databases of the university hospitals of Cologne and Muenster, and were asked, via phone call more than 24 h prior to survey execution, for their willingness to participate in the study. Important inclusion criteria were adult age (of 18 or more years), German language as mother tongue, histologically confirmed diagnosis of cerebral glioma WHO grade 3 or 4, as well as the absence of heavily disabling communicative or other neurocognitive deficits (thus impeding the informed consent and/or an evaluable self-report). Further demographic characteristics such as gender and educational level were assessed but did not represent in-/exclusion criteria.

#### 2.1.3 Speech language therapists

Target professionals, i.e., speech and language therapists with professional and practical expertise, were recruited in September and October 2022 via professional and social networks, as well as personnel contacts of the authors, and were asked to participate in the survey anonymously. Here, we aimed at the collection of data from at least 20 respondents.

### 2.2 Survey components and administration

In a paper-pencil survey, which was distributed by mail, the existence of technical equipment, the availability of a household member or other well-known person with substantial computer and internet skills, as well as the participants' own computer and internet skills were assessed, along with the motivation to participate in a telemedicine assessment or therapy. Moreover, age, gender, and educational level were included in the data base.

#### 2.2.1 Survey of healthy participants

At the beginning of the survey, healthy participants were instructed to imagine themselves in a situation of severe illness (Imaginary patients' perspective; cf. Supplementary material for wording and details).

#### 2.2.2 Survey of patients

In the HGG patients' survey version, the following additional parameters were assessed: mobility, histological grade, and residency-related parameters i.e., number of inhabitants, public transport facilities, as well as distance from the closest centre for integrated neuro-oncology and from the treating neuro-oncological care unit (cf. Supplementary material for wording and details). Of note, the last-mentioned parameters were assessed by the authors according to the postal code of the participants' residency and of the respective healthcare centres.

Finally, the overall clinical status of the patients was also considered, using a binary scale to describe whether the patients were physically or mentally incapacitated to the extent that they could not care for themselves [according to a Karnofsky Performance Index (KPI; Karnofsky and Burchenal, 1949) of 70–100/100 according to the latest documented medical assessment].

#### 2.2.3 Survey of speech and language therapists

The assessed parameters of the conditionally programmed electronic survey included caseload characteristics (i.e., the relative number of patients seen by therapists with a diagnosis of HGG vs. other acquired neurogenic communication disorders), and the frequency of use of telemedicine and other digital technologies in the assessment and/or therapy of acquired neurogenic communication disorders. Both the type of the digital technology and the type of communication disorder were asked to be further specified.

Furthermore, speech and language therapists were asked to rate the extent to which they believed that telemedical vs. in-person settings were therapeutically equivalent, which patient subgroups might be more vs. less suitable for telemedical approaches, and how digital participation could be improved.

Regarding brain tumour patients, speech and language therapists were also asked if they consider the referral rate for the therapy of communication disorders sufficient and, if not, to speculate over possible reasons.

Moreover, age, gender, and educational level, as well as the professional qualification and work environment of the survey participants were included in the database.

### 2.3 Statistical analysis

The statistical analysis was performed using LibreOffice Calc (version 6.4.7.2) and R Studio (version 2023.06.1, based on R version 4.3.1). The statistical threshold was set to *p* < 0.05 by default. Notably, data distribution was considered not normal if the Shapiro-Wilk test resulted in a significance estimation of *p* < 0.01. Whenever appropriate, false discovery rate (FDR) correction was applied for multiple comparisons (Benjamini and Hochberg, 1995).

Propensity score-based, pairwise (1:1) matching of control subjects to the HGG patients' cohort was performed using the “optmatch” package in R and the {matchit} function (Hansen and Klopfer, 2006). The underlying algorithm uses the sum of the absolute pair distances between the respective control units and the corresponding treated units in the matched sample, similar to the nearest-neighbour matching method. This procedure automatically allocates the best matching partner out of the control group to each patient (not allowing for multiple allocation of controls), in this case considering the three major demographic parameters age, gender, and educational level (binary scale with “high” defined by holding a university entrance qualification) as matching criteria. The resulting matched control group is referred to as Propensity-Score-Matched-control (PSM-control) group throughout the manuscript.

For group comparisons between binary-scaled data, Fisher's exact test was used, whereas for comparisons between metric variables, Student's *T*-test or Wilcoxon's rank sum test was applied (for normally distributed vs. non-parametric data, respectively).

To evaluate the factorial influence on the motivation for telemedicine participation, the factors motivation and skills were converted into four-level ordinal scales, ranging from 0 (not motivated/unskilled) over 1 (unsure) and 2 (pandemic-dependent motivation/household member skilled) to 3 (generally motivated/own skills). For correlations with continuous or ordinal parameters, Spearman's rank correlation coefficient (rho) was calculated. For associations with binary-scaled parameters, point-biserial correlations (corr) were computed. The following factors were considered for multiple correlations analysis: age (continuous), gender (binary), educational level (binary), the presence of complete technical equipment (binary), the availability of skills in the household (four levels), patients' car mobility (binary), dependency on a driver (binary), size of the town/city of residency (continuous), distance between residency and treating neuro-oncological unit (continuous), and the self-caring clinical status (binary).

Moreover, a multivariate ordinal regression analysis was performed using the {vgm} function for vector generalised linear model fits from the “VGAM” package (Yee and Wild, 1996; Yee, 2015) in R. To this end, feature selection was performed based on the results of the aforementioned correlation tests, using a liberal statistical threshold of *p* < 0.2. To achieve homogeneous scale levels among included factors, the selected non-binary features were binarised according to the group median.

To explore and visualise the distributions of age and travel distance (from home to the specialised treatment unit) across motivational levels, violin plots with added scattered dots were created using the “gglot2” library (Wickham, 2016) and the {geom_sina} function of the “ggforce” library in R (Pedersen, 2022).

## 3 Results

### 3.1 Readiness of HGG patients vs. PSM-controls for telemedicine

#### 3.1.1 Participants

Seventy-three healthy subjects (47% males, 53% females) were surveyed, most of them with high educational level (62% graduated with a university entrance qualification) and with a median age of 64 years, ranging from 24 to 95 years.

In addition, 56 HGG patients (age median: 59 years; range: 21–88 years; 48% males/52% females; 45% with a university entrance qualification) including 91% glioblastoma patients (WHO grade 4) and 9% patients with anaplastic glioma (WHO grade 3) were included in the study.

To balance the patients' age, gender, and educational level against a matched cohort of healthy subjects, two equally sized cohorts of patients and healthy subjects (56 subjects each) were composed by pairwise matching using the aforementioned propensity-score matching procedure (cf. methods, statistics). The respective demographic characteristics of the patient cohort and the resulting PSM-control group (with an age median of 60 years, 50% males/females and 54% of high educational level) are summarised in the top section of Table 1.

**Table 1:** Overview of demographic characteristics and survey results of HGG patients vs. propensity-score-matched (PSM-) controls.

**Demographic characteristics**	**HGG patients**	**PSM-controls**	**Fisher exact/*T-test***
Age (median [range])	59 [21;88]	60 [25;92]	*p = 0.544*
Male gender	48%	50%	*p* = 1
High educational level	45%	54%	*p* = 0.450
**Technical equipment**			
Computer or tablet	95%	88%	*p* = 0.527
Computer	93%	84%	*p* = 0.237
Tablet	46%	54%	*p* = 0.255
Web-camera	63%	64%	*p* = 1
Headset	45%	34%	*p* = 0.333
**Computer/internet skills**			
Own skills	57%	52%	*p* = 0.705
Skills of household member	36%	48%	*p* = 0.251
Unsure	11%	20%	*p* = 0.292
No skills in household	11%	11%	*p* = 1
**Motivation**			
Generally motivated	54%	41%	*p* = 0.256
Motivated due to pandemic	14%	16%	*p* = 1
Unsure	18%	27%	*p* = 0.364
Unmotivated	14%	16%	*p* = 1
**Mobility (trip to medical appointment)**			
Driving own car			
Possible	18%		
Regularly chosen option	14%		
Driven by household members	61%		
Taxi	20%		
Bike/walking	20%		
Public transport	4%		
**Clinical status**			
Self-caring ability (physical and cognitive)	86%		
**Logistics (residency** ↔**treating medical unit)**			
Inhabitants (median [range])	23,145 [1,357;1,088,040]		
**Distance (median [range])**			
To closest neurooncological unit	20 [0;73]		
To treating neurooncological unit	32 [0;402]		
**Public transport facilities of residency**			
Railway	80%		
Bus terminal	75%		
Bus stop	98%		

The table provides the data of the two equally sized groups (n = 56 each). Of note, mobility data (related to the treatment-coordinating hospital) were not obtained from the PSM-control subjects.

#### 3.1.2 Practical prerequisites for digital participation

The survey results show that, overall, appropriate equipment for digital participation is widely available in private households. Among HGG patients, 93% of the participants have access to a computer, and the majority (63%) possess an additional web camera, whereas more than a third of the participants stated having a headset available (45%).

Beyond the technical facilities, more than half of the survey participants stated that they felt sufficiently comfortable themselves with internet-related applications (57%), and the vast majority reported sufficient internet skills in their household (79%). However, some participants (11%) were unsure about the availability of sufficient skills, and 11% reported no appropriate digital experience in their household. No statistically significant group differences were found between the patient and the PSM-control cohort for any of these criteria (Table 1).

#### 3.1.3 Motivation for telemedical participation and related factors

Again, no significant difference was observed regarding the response behaviour of both groups (cf. Table 1 for groupwise results and comparative statistics). Approximately half of the patient survey participants (54%) reported a general willingness to participate in telemedical assessments or interventions, also beyond the context of a pandemic. Another 32% of the respondents would consider participation driven by a pandemic or were otherwise unsure. The minority (14%) claimed to be unmotivated for digital participation in the medical context.

To investigate the influence of various factors on the motivation for telemedical participation, correlation analyses were performed separately for both the HGG patient and the PSM-control cohorts.

**Figure 1:**
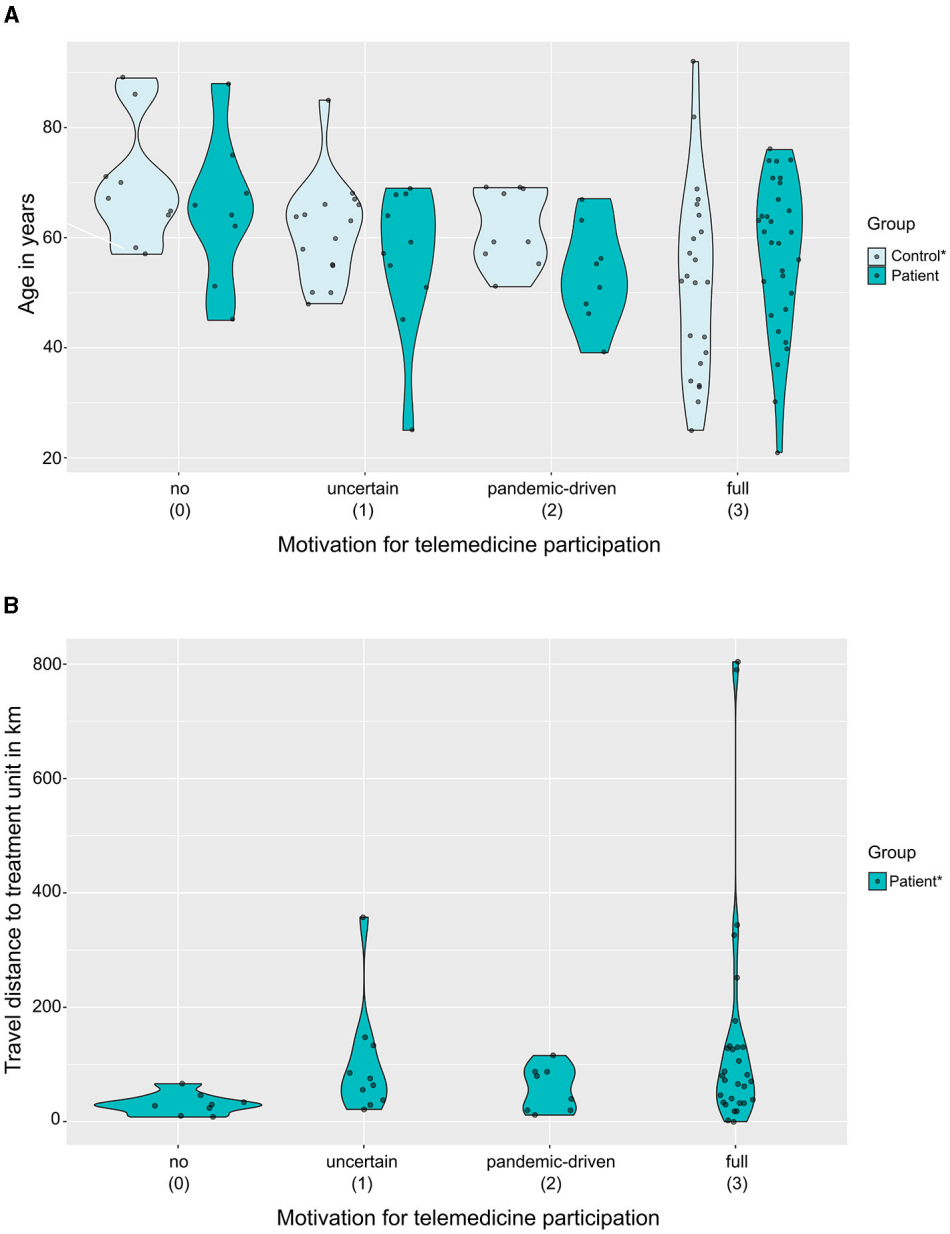
Distribution of age and the distance of the treating neurooncological unit from home, grouped by motivational levels. The violin plots with added scattered plots illustrate the relationship of the motivational levels (x-axis) with **(A)** age (y-axis), grouped by patient (blue) vs. PSM-control (light blue) cohorts; and with **(B)** the travel distance from home to the neuro-oncological treatment unit of the HGG patients. *Significant Spearman's correlation: *p* < 0.05.

In **HGG patients**, neither age (rho = −0.08; *p* = 0.535), gender (*p* = 1), or education (*p* = 0.450) nor availability of skills in the household (corr = 0.05; *p* = 0.689) significantly influenced motivation. For this cohort, significant correlations were found with not self-caring clinical status (ordinal variable; rho = −0.29, *p* = 0.030) as well as with the distance of the residency from the treating neuro-oncological unit (metric variable; rho = 0.28, *p* = 0.035), corresponding to a significant difference between the patients with long vs. short distance (i.e., </≥33 km; *p* = 0.048). Moreover, a statistical trend was observed for the availability of full technical equipment (corr = 0.22; *p* = 0.101). In contrast, for the **PSM-control cohort** (imaginary patients' perspective), significant associations were found with the factors age (metric variable; *p* = 0.002, rho = −0.41), equipment (*p* < 0.001) and skills (*p* < 0.001), whereas no significant effect was apparent for the factors gender (*p* = 0.382) and education (*p* = 0.596). Correlations of motivational level with binary or binarised factors reaching the level of at least statistical trends are summarised in Table 2. For an additional visual impression of the distributions of age and travel distance across motivational levels, see Figure 1.

**Table 2:** *N*-fold cross table of significant factors by motivational level.

**Cohort**	**Factor**		**Motivational level**				**Corr. rho (uncorrected *p*-value)**
			**0 un-motivated**	**1 unsure**	**2 motivated in pandemic**	**3 generally motivated**	
PSM- controls	Complete technical equipment	Yes	0	2	1	15	0.55^***^ (1.3 × 10^−5^)
		No	9	13	8	8	
	Adequate skills in household	Yes	2	9	9	21	0.54^***, $^(1.5 × 10^−5^)
		No	7	6	0	2	
	Age (years)	≥60	7	9	4	8	−0.31^*, §^(0.018)
		< 60	2	6	5	15	
HGG patients	Self-caring clinical status	Yes	8	10	7	23	−0.29 (0.028)
		No	0	0	1	7	
	Close distance to neuro-oncological unit (km)	< 33	7	5	4	12	−0.28 (0.034)
		≥33	1	5	4	18	
	Complete technical equipment	Yes	2	1	3	15	0.22 (0.101)
		No	6	9	5	17	

Of note, for the binarisation of ordinal or metric factors, cut-offs were set to the group median, i.e., travel distance of 33 km, age of 60 years, and skills: adequate own digital skills or respective skills of household member.

Corr, Pearson's point-biserial correlation; PSM, Propensity score based matched.

Grey font: weak statistical trend, 0.05 < p < 0.2.

^*^p < 0.05 (FDR-corr.), ^***^p < 0.001 (FDR-corr.).

^$^Correlation with ordinal variable of household skills (four levels): p = 4 × 10^−8^ (i.e., p < 0.0001, FDR-corr.).

^§^Correlation with metric variable age: p = 0.0015 (i.e., p < 0.05, FDR-corr.).

Based on the results of the correlation analysis and applying a threshold of *p* < 0.2 for feature selection, multifactorial ordinal regression analysis was performed including the remaining binary/binarised factors (see Table 2) and highlighted that the strongest influence expressed by the odds was attributed to the availability of complete technical equipment for both cohorts (PSM-controls: odds = 9.6 vs. HGG patients: odds = 2.1; Table 3). The supposed age effect in the PSM-control cohort proved non-significant in the multivariate analysis (*p* = 0.536; Table 3). However, at least for PSM-control subjects, an additional effect of the in-house availability of internet skills was shown (odds = 2.8, *p* = 0.005). Although not reaching the level of statistical significance, the HGG patients' travel distance from home to the medical treatment unit and the overall clinical status contributed to the model, with a comparatively higher odds being attributed to the clinical status (odds = 0.2 vs. 0.4; Table 3). This may reflect that the vast majority (88%) of patients in incapacitated clinical status were generally motivated for a telemedicine participation (cf. Table 2).

**Table 3:** Ordinal regression analysis to evaluate factorial influence on the degree of motivation for telemedicine participation.

**Factor**	***z*-value**	***p*-value**	**Odds**
**PSM-controls**			
Age ≥ 60 years	0.6	0.536	0.7
Complete technical equipment	3.0	0.003^**^	9.6
Adequate skills in household	2.8	0.005^**^	7.4
**HGG patients**			
Self-caring clinical status	−1.6	0.108	0.2
Distance residency—treating neurooncological unit < 33 km	−1.5	0.136	0.4
Complete technical equipment	1.3	0.211	2.1

Of note, distance, age, and skills level were included as binarised measures (cf. Methods and Statistical analysis).

^***^p < 0.001.

### 3.2 Survey of speech and language therapists

#### 3.2.1 Participants

Twenty-three female speech and language therapists, mostly of young age (61% <35 years; 9% 35–39 years; 4% 40–44 years; 13% each: 45–49 and 50–54 years) participated in the survey. the vast majority held a university diploma/master's degree (87 %), and approximately a third of the participants worked in either an outpatients' clinic (39%), a rehabilitation centre (30%), or an acute care hospital (35%), and 13% in a nursing home. Thirteen percent of the participants were additionally affiliated to an educational institution (professional school or university), and 17% were self-employed.

#### 3.2.2 Digitisation in acquired neurogenic communication disorders

The vast majority of the participants (91%) stated that they regularly worked with individuals diagnosed with neurogenic communication disorders (i.e., at least 15% of their case load). Only 9% of the respondents reported encountering neurogenic communication disorders very rarely (i.e., in <1% of the consultations) in clinical practise.

A third of the participants (35%) responded that they used telemedicine and other digital instruments or procedures for the diagnostics or therapy of neurogenic communication disorders. Most of them (88%) apply telemedicine and other digital tools for aphasia therapy, with half of them using them regularly and the other half on an exceptional basis (i.e., in <5% of the therapy sessions). Of note, the regular use of video conferences in this context was unusual (13%).

Regarding the equivalence of the diagnostic/therapeutic value of video conferences respectively, 75% stated that the telemedicine setting would yield worse results than an in-person setting, whereas the remaining 25% assumed equality.

There was a broad consensus among survey participants that certain patient characteristics are particularly (un)suitable for a telemedicine diagnostic/therapy setting for neurogenic communication disorders. For instance, elderly and severely incapacitated patients were widely regarded as unsuitable by several participants, whereas young and cognitively fit patients, were assumed to be well-suited, especially if they live in rather remote places and/or have limited mobility (Table 4). Interestingly, distinct survey participants added that they regard video-based remote intervention particularly useful to increase the frequency of therapy sessions, also in the context of returning to work, rather than to fully replace the in-person setting. Others pointed out that patients with dysarthria and (chronic) aphasia without major additional cognitive deficits might represent a rather good target for a telemedicine therapy setting. Likewise, one participant suggested the Lee Silverman voice treatment (LSVT) for Parkinson's disease as a particularly well-suited telemedicine application.

**Table 4:** Presumed suitability of patients for telemedicine.

**High suitability**		**Low suitability**	
**Characteristics**	***N*** **(proportion)**	**Characteristics**	***N*** **(proportion)**
Good cognitive performance	6 (43%)	Advanced age	8 (42%)
Reduced mobility/necessity of medical home visits	6 (43%)	Cognitive deficits	8 (42%)
Long travel distance	5 (36%)	Poor technical skills/resources	8 (42%)
Young age	4 (29%)	Strong deficits (e.g., receptive aphasia and speech apraxia)	7 (37%)
Good technical skills/resources, affinity with media	4 (29%)	Vision/hearing deficits	3 (16%)
Poor general health condition	2 (14%)	Poor general health condition	2 (11%)
Supporting household member	2 (14%)	Others: dysphagia, non-native speakers, poor collaboration, and nursing home residents	1 (5%) each

The table summarises the essence of the free text entries made by the surveyed therapists.

#### 3.2.3 Experience with brain tumour patients

The majority of the participants (73%) have experience with the diagnosis/treatment of communication disorders in brain tumour patients; however mostly rather sporadically. Only four participants responded that brain tumour patients constitute at least 20% of their caseload. Of note, most of these participants responded that in their experience, the referral of brain tumour patients to speech/communication therapy happens either too late (91%) or insufficiently (64%). The survey participants suspected that lack of knowledge/awareness of the therapeutic potential, lack of time/workload, as well as different priorities (e.g., tumour control) of the medical staff could be important reasons for inappropriate referral.

Interestingly, 78% of the survey participants expressed the opinion that digital participation of brain tumour patients in the diagnostics and therapy of communication disorders should be improved. They suggested enhancing access and introduction to digital media and participation, as well as developing specific apps for individualised speech, language, and communication training (e.g., for self-instructed therapy) as possible ways to achieve advancements in this context.

## 4 Discussion

The results of our surveys show that telemedicine applications could have great, still largely unexplored potential for the treatment of brain tumour patients. The increased use of digital technologies could help to close the diagnostic and therapeutic gaps in this particular, heavily burdened patient group and spare them long and exhausting journeys to follow-up examinations (eventually without pathological findings). In the therapeutic setting, for example, the diagnosis and therapy of speech, language, and communication disorders in brain tumour patients could be improved and the frequency of treatment increased.

Although only a minority of German speech and language therapists currently use telemedicine or other digital diagnostic or treatment tools on a regular basis (in- or outside the context of brain tumour patients), there is a broad consensus that improving the participation of this patient cohort through telemedicine and other digital technologies is worthy of further attention and enhancement.

The vast majority of the interviewed HGG patients was open to digital technologies, was overall well-equipped, and felt sufficiently skilled to participate in video-based telemedical assessments and interventions. Only a few of all interviewed subjects (15%) refused such receptiveness. In large agreement with the assumptions expressed by the participating speech and language therapists and our initial hypotheses, the factorial analysis of the HGG patients' survey showed that digital offers would be of particular interest to patients in reduced general health condition and those living in rather remote locations, far from centralised treatment offers. Interestingly, the particular receptiveness of these patient subgroups to digitisation seemed to outweigh the effects of age, equipment, and internet skills, which were only evident in the PSM-control cohort.

### 4.1 Openness to telemedical participation: imaginary scenario vs. “real-life”

Our hypothesis that the “real-life” HGG patients' perspective might render subjects more receptive to digital participation compared to imaginary severe disease, i.e., healthy subjects imagery perspective, was not proven in this study. This is possibly due to the sample size, which was not designed to show (rather) weak statistical effects. Although we observed a higher proportion of “unsure” subjects in the PSM-control cohort (27% vs. 18%) against a higher percentage of generally motivated patients compared to PSM-controls (54% vs. 41%), these differences were not statistically significant. On the other hand, the lack of significant differences in response behaviour may suggest that the imaginary disease instruction of PSM-controls could be an adequate model to assess telemedicine readiness and, thus, be helpful to accelerate future surveys of this type by prioritising more readily available healthy cohorts.

### 4.2 Availability of technical equipment, digital skills, and support

The availability of technical equipment at home and corresponding knowledge, possibly conveyed by a relative or friend, are fundamentally conducive to participation in telemedicine offers. Across both PSM-control and patient cohorts, this survey demonstrated a good overall availability of the basic technical equipment, with >90% possessing a computer or tablet and approximately two-thirds of people being equipped with a webcam. The results are comparable to the results of the SIM study (Medienpädagogischer Forschungsverbund Südwest, 2021), a comprehensive investigation of the media use of people over 60 years of age in Germany, although our survey showed even higher rates of appropriate equipment (computer, tablet or similar). A possible explanation for this difference could be the high proportion of people with higher educational qualifications in our survey. Another encouraging aspect of this study is the largely positive assessment of one's own abilities or the abilities of a person in one's own household, enabling the use of telemedicine offers. In contrast, only 11% of the survey participants denied having adequate computer and internet skills in the household, which largely overlapped with those participants lacking technical equipment. As a result, ~90% of the target population, including a large proportion of patients in advanced age groups, is technically eligible for telemedicine offers. This highlights the great potential and expediency of expanding telemedicine applications for brain tumour patients.

### 4.3 Effects of demographic and disease-related factors on motivation for digital participation

In addition to technical resources and skills, willingness to accept telemedicine offers are also decisive for their successful implementation in clinical practise. In this study, approximately half of the respondents were intrinsically motivated regarding telemedical services such as video-based assessments and therapy, making them readily available. Another third were unsure or linked their motivation to the framework conditions of a pandemic. Although the principle problem of an unacceptable underrepresentation and under treatment of communication disorders (and other neuropsychological and neurocognitive disorders) in brain tumour patients remains widely unchanged after the pandemic, it seems noteworthy that the pandemic, despite its myriad of negative impacts, seems to have significantly improved access and openness to participation through digital technologies (Cacciante et al., 2022; Jaecks and Jonas, 2022) among people and professionals of all generations. This is partly reflected by the 15% of survey respondents who reported their motivation as being driven by the pandemic.

Especially among this undecided patient group, better information about the possibilities and requirements of telemedical tools, not only as stand-alone assessments/interventions but also as a practical complement to the traditional, in-person setting, could help to increase willingness to participate. Only 15% reported being unmotivated, which again largely overlapped with survey participants lacking technical equipment and/or in-house skills. Conversely, up to 85% of the target group showed at least a possibly positive attitude towards medical digitisation. At first glance, surprisingly, and in contrast to both our initial assumptions and the results obtained from PSM-controls, neither age nor any other assessed demographic factor played a significant role in the extent of motivation towards telemedicine participation among HGG patients. This could be due to the strong effects of other parameters, especially non-independent general health status, which may have significantly outweighed weaker effects in the patient cohort. In other words, the real-life challenges related to disability and the efforts related to travelling might render even elderly and less well-equipped or skilled patients interested in telemedicine interventions.

The key findings of the factorial analysis, however, i.e., a significantly higher motivation level among considerably incapacitated patients and, as a trend, among patients with a long distance between home and neuro-oncology treatment unit, are very much in line with our initial assumptions and underline the need for investment in digitisation. Our data may reflect the fact that HGG patients with high rehabilitation needs are unfortunately the least accessible due to their limited mobility. Furthermore, the results suggest that regions with a rather thin network of available treatment units might be particularly worthy of introducing telemedicine assessments and interventions.

### 4.4 Status-quo of incorporating digitisation into clinical practise: between opinion, experience, and innovative ideas

Although there is growing agreement that digital technologies can be helpful, especially to people with disabilities, digitisation in the field of neuro-oncology is still in its beginnings. Even among the responding speech and language therapists, there is a broad consensus that participation of brain tumour patients through digital technologies in the diagnosis and therapy of speech, language, and communication disorders should be improved. Despite the great awareness of the special needs of patients with limited mobility, poor general condition and long journeys to the therapist, the proportion of speech and language therapists who use telemedicine or other digital tools on a regular basis is very low. In this context, it seems appropriate to point out that only one of the participants indicated having personal experience with video-based telemedical interventions.

The lack of regular use of telemedicine and other digital technologies in our sample of speech and language therapists thus could be caused by lack of personal experience with these technologies in daily practise. Also, a general scepticism about the use of digital technologies in speech and language therapy due to concerns about being replaced by them as well as the assumptions of an assumed lack of suitability for patients of advanced age (Jaecks and Jonas, 2022) could be reasons for non-application. The latter in particular could not be proven in our patient survey data. Another factor seems to be the assumption of 75% of the surveyed therapists that, in a digital setting, both the reliability of diagnosis and the improvements achievable by therapy are inferior to the outcome of an in-person setting. It seems obvious that teletherapy is not the treatment of choice for elderly patients with brain tumours and presumed language-independent cognitive deficits. However, the recent data on the equivalence of in-person vs. telemedical or otherwise digital (diagnostics and) therapy of language, communicative, and cognitive functions do not paint a uniform picture (Kester, 2020; Weidner and Lowman, 2020). Research from the times of the COVID-19 pandemic regarding the this question suggests that the more similar in-person and digital approaches are in terms of, for example, input and output modality or other surrounding factors (special equipment needed; need of e-helpers), the more likely it is that the approaches are equivalent, and that results and effects are comparable (Kester, 2020). In the context of blended teaching concepts, e.g., based on online e-learning, have even proven to achieve better knowledge outcome than traditional strategies (Vallée et al., 2020). These surprisingly clear meta-analytic results might point towards a similarly great potential of blended therapy concepts, e.g., in the context of speech and language therapy. Likewise, recent evidence has been provided regarding the implementation telemedicine among speech and language therapists working with adults with neurogenic communications disorders (e.g., Cacciante et al., 2021; Gherson et al., 2023; Teti et al., 2023).

An important point noted by several responding speech and language therapists is that digital devices alone can hardly replace the in-person setting. However, they can be a great help in providing access to therapy for patients who would otherwise not have access to services and in enabling increased treatment frequency or consolidating treatment effects, e.g., through self-instructed repetition of defined tasks.

Finally, it seems important to distinguish between fully automated/self-directed assessment or treatment tools and video-based telemedical conferences, which offer far more interactive possibilities and make the telemedical intervention almost in-person, providing a major advantage for the two most vulnerable patient groups: patients in poor general health and those living far away. On the other hand, self-instructed tools save human resources on the part of therapists and can thus partly compensate for the imbalance between demand and supply of professional treatment in this field.

Other major challenges for speech and language therapy in HGG patients compared to other cohorts include time management, treatment priorities, and the psycho-oncological burden of the disease. The authors agree with the opinion of some responding speech and language therapists that all these parameters might contribute to the fact that the referral rate of brain tumour patients with communication disorders to appropriate therapy is largely insufficient. Patients with malignant brain tumours are usually overloaded with radio-oncology and neuro-oncology appointments, especially in the early phase after diagnosis, often accompanied by psycho-oncological interventions, physiotherapy and/or occupational therapy sessions due to third party burdens/deficits. In addition, many of them are not allowed to drive due to a clinical history of epileptic seizures, which makes the repeated in-person medical and therapy visits in many cases a major challenge. Therefore, telemedicine interventions might be particularly well-suited to improve access to speech therapy while addressing the special needs of HGG patients.

### 4.5 Limitations

This study summarises the opinions and experiences as well as the technical possibilities of both the target group (of HGG patients) and the speech and language therapists in order to shed light on the opportunities and obstacles linked to telemedical applications in speech and language diagnostics and therapy of those patients. Unfortunately, due to the anonymous character of the surveys and our attempt to limit the survey length to the necessary minimum in order to achieve a high compliance rate and avoid missing values, only very little clinical data have been obtained from the interviewed patients. Additional information, for instance on the nature and the degree of eventual communication impairments or on the status of therapy and disease, would have been of great value to widen the field of interpretation of the survey results.

Moreover, it must be considered that the interview statements of healthy controls, patients and therapists might not readily translate to the effective daily clinical practise, which reflects a common assumption in market research. Accordingly, previous studies in the field like the BIG CACTUS randomised-controlled trial have demonstrated that positive effects of digital treatment strategies might (i) not necessarily improve daily-living communication skills or health-related quality of life (Palmer et al., 2020), and might (ii) be biased by the therapists' and the patients' attitude and knowledge regarding the implementation of digital technologies into therapy practice (Burke et al., 2022).

Another major limitation of the surveys is the relatively limited number of participants, linked to a certain regional bias, as the HGG patients and probably also the therapists were mainly recruited from the mid-west of Germany (a highly developed region with a relatively dense network of medical and speech and language therapy services) due to the affiliations and the regionally focused professional network of the authors.

Thus, the data are not sufficiently powered to demonstrate small effects and might not be readily transferable to a different socio-geographical background. In the authors' opinion, however, the core message of a profound need for expanding telemedical infrastructure and access might be rather underestimated due to the assumed socio-geographical bias.

### 4.6 Outlook

Given the encouraging openness of HGG patients to telemedicine and the broad consensus among the therapists that the access to speech and language therapy and digital participation in this context need improvement, it seems timely to develop strategies for such progress. Efforts may involve public and social media, patient, caregiver, and therapist (professional) organisations and networks to raise awareness and increase openness for telemedical and other digital assessment/therapy approaches by highlighting the wide variety of possible digital solutions and critically discussing their respective benefits and limitations. Moreover, it seems advisable for researchers and interested clinicians in the field to join forces in order to further elaborate on the available spectrum of tools that enable user-friendly telemedical and other digital assessment/therapy options tailored to the target patient population. In this context, it might be of great advantage to achieve non-commercial and free release of the respective tools. However, not only the recent legislative advances regarding data safety issues as well as the subjection of medical software products to approval for clinical use, but also the increasing automatation of the respective tools, turn their design and validation process so complex and cost-instensive that a purely non-commercial development has become challenging.

Such efforts could not only represent a major advance in the treatment of language and communication impairments *per se* but could also be used to facilitate other medical applications such as a more comprehensive and regular testing of neurocognitive functions, which is currently rarely implemented in routine clinical practise (Weiss Lucas et al., 2021). Early detection of cognitive decline in HGG patients may not only help to better identify treatment/support needs, but also to anticipate suspected tumour recurrence (Armstrong et al., 2003; Meyers and Hess, 2003; Butterbrod et al., 2019), and thus possibly even improve the survival outcome of those patients.

## 5 Conclusion

The morning after the pandemic leaves us with the reverberations of the increasing acceptance of telemedical tools that can be sensed across generations from the response behaviour of the surveys presented here. However, the great potential that lies in amending digital technologies to the in-person setting seems to have hardly found its way into the daily practise of speech language therapists, thus leaving the substantial problem of under detection and under treatment of speech and language disorders in brain tumour patients widely unchanged. Achieving a paradigm shift in this context may be particularly important for HGG patients who not only struggle with neurogenic speech, language and communication disorders, but also with mobility and time limitations due to the impact of further neurological deficits as well as the side effects and time schedule associated with tumour treatment. An expansion of accessible (and ideally free) telemedicine applications could serve as crucial support, empowering the therapists' answer to the currently underestimated need for assistance. Therefore, joint efforts to newly develop and further improve digital tools tailored to this patient population seem timely and worthwhile.

## Data availability statement

The raw data supporting the conclusions of this article will be made available by the authors upon reasonable request, without undue reservation.

## Ethics statement

The studies involving humans were approved by Ethics Committee of the Medical Faculty of the University of Cologne (File No. 14-109). The studies were conducted in accordance with the local legislation and institutional requirements and according to the guidelines for good clinical practise as well as with the Delaration of Helsinki. Written informed consent for participation was not required from the participants or the participants' legal guardians/next of kin because, the data consist of anonymous survey data. According to legislation and its interpretation by the Local Ethics Committee, no written informed consent is necessary in this case. None of the participants received financial compensation for the participation in the survey.

## Author contributions

CW: Conceptualisation, Data curation, Formal analysis, Investigation, Methodology, Supervision, Visualisation, Writing – original draught, Writing – review & editing. SK: Conceptualisation, Investigation, Writing – review & editing. JJ: Investigation, Writing – review & editing. RL: Investigation, Methodology, Writing – review & editing. MK: Methodology, Writing – review & editing. RG: Resources, Writing – review & editing. DW: Conceptualisation, Investigation, Writing – review & editing. KJ: Conceptualisation, Investigation, Methodology, Resources, Supervision, Writing – original draught, Writing – review & editing.
