# Supplementary material for: Digital participation of brain tumour patients in the assessment and treatment of communication disorders
Source: Front Psychol. 2024 Jan 8;14:1287747. doi: 10.3389/fpsyg.2023.1287747 (PMC10800882; doi:10.3389/fpsyg.2023.1287747)
Supplement: Supplementary file 1 [file Data_Sheet_1.PDF]

SUPPLEMENT

Surveys of healthy subjects and brain tumour patients

Instruction for healthy subjects:
We are currently conducting a survey project on digital participation in brain tumour patients. To better understand the results, we also need information from healthy controls. Please help us by answering the questions below. Thank you very much! Your data will remain completely anonymous! However, we would like to know your age, your gender and your school qualification level.

Instruction for brain tumour patients:
Do you suffer from a higher grade glioma (grade III or grade IV)? Then you can help us plan a scientific project! Please answer the following questions. Thank you very much in advance! Your data will remain completely anonymous! However, we would like to know your age, your gender and your school qualification level.

Common survey questions [minor adaptations for patients / for healthy subjects]:

Age: _____ years
Gender: □ male / □ female / □ diverse
Highest-ranking school leaving degree / diploma: ____________

Q1 Availability of computer (equipment)
Do you have one or more of the following available to you at home?
(Please tick as appropriate - multiple ticks possible)

- ☐ Computer/laptop
- ☐ Tablet
- ☐ Computer camera (suitable for video conferencing)
- ☐ Headset (suitable for videoconference)

Q2 Availability of related parties
Do you have a friend or relative (in your household or as a regular visitor)? Could they provide your doctors with information about your state of health (provided you wish to do so)?
(Please tick as appropriate)

- ☐ Yes (in the same household)
- ☐ Yes (not in the same household)
- ☐ No

From now on, please assume the following: You would have all the equipment mentioned in question 1 available at home.

Q3 Computer/Internet skills
There is the possibility to participate in internet-based videoconferences with medical doctors.
Would you or a friend or relative (as mentioned above) be able to participate?
(Please tick as appropriate - multiple ticks possible)

- ☐ Yes (myself)
- ☐ Yes (my friend or relative)
- ☐ Maybe / I am not sure
- ☐ Not at all

Q4 Motivation

Suppose that medical examinations (e.g. for progress assessment) and interviews (e.g. on support needs) regarding your brain tumour can also take place video-based from home instead of at the neuro-oncological tumour centre.

[Variant for healthy subjects: Medical examinations (e.g. to assess progress) and interviews (e.g. on support needs) can also take place video-based from home. Imagine that you undergo regular treatment and follow-up visits by a medical doctor due to a serious illness!]

Would you make use of such an offer?
(Please tick as appropriate)

- ☐ Yes (during a pandemic)
- ☐ Yes (also independent of a pandemic)
- ☐ Maybe / not sure
- ☐ Definitely not

Questions for brain tumour patients only:

Q5 Mobility

Q5a Do you have a car driving license?

- ☐ Yes
- ☐ No

Q5b Do you own a car?

- ☐ Yes
- ☐ No

Q5c Do you feel physically able to drive (even if you are not currently allowed to)?

- ☐ Yes
- ☐ No

Q5d Are you currently allowed to drive?

- ☐ Yes
- ☐ No

Q5e How do you mainly get to your doctor's appointments?

- ☐ On foot
- ☐ By bicycle
- ☐ By car: driving myself
- ☐ By car: driven free of charge by another person
- ☐ By taxi
- ☐ By public transport
