# Supplementary material for: Digital participation of brain tumour patients in the assessment and treatment of communication disorders
Source: Front Psychol. 2024 Jan 8;14:1287747. doi: 10.3389/fpsyg.2023.1287747 (PMC10800882; doi:10.3389/fpsyg.2023.1287747)
Supplement: Supplementary file 2 [file Data_Sheet_2.PDF]

Survey of Speech language therapists

Structure and essential questions of the survey. Of note, very minor adjustments were made to simplify the presentation of the conditionally programmed survey.

Section 1 Participant characteristics

Q1 Age:

- ☐ <35 years
- ☐ 35-39 years
- ☐ 40-44 years
- ☐ 45-49 years
- ☐ 50-54 years
- ☐ 55-59 years
- ☐ 60 years or older

Q2 Educational level:

- ☐ University entrance qualification + other specific education
- ☐ Bachelor title or equivalent (e.g. technical college degree)
- ☐ Master title or equivalent (University diploma)
- ☐ Doctoral degree

Q3 Work environment:

- ☐ Outpatients clinic
- ☐ Rehabilitation unit/hospital
- ☐ Hospital (acute care)
- ☐ School
- ☐ University

Q4 Employment

- ☐ Employed
- ☐ self-employed

Section 2 Digital instruments/procedures in acquired neurogenic speech/communication disorders

Q5 How often do you treat or diagnose acquired neurogenic speech/communication disorders in your everyday practice?

- ☐ Never
- ☐ Very rarely (< 1%)
- ☐ Rather rarely (1-14%)
- ☐ Sometimes (15-24%)
- ☐ Rather often (25% - 49%)
- ☐ Frequently (50% or more)

Q6 Do you use digital instruments or procedures in the diagnosis and/or therapy of acquired neurogenic communication disorders?

- ☐ No
- ☐ Yes, diagnostics
- ☐ Yes, therapy
- ☐ Yes, both diagnostics & therapy

Q7 For which types of disorder? [→ Free text entry]

Q8 In which proportion of your patients with acquired neurogenic communication disorders do you use the digital instruments or procedures?

- ☐ Never
- ☐ Rarely (< 5%)
- ☐ Rather rarely (5-19%)
- ☐ Rather often (20% - 49%)
- ☐ Frequently (50% or 89%)
- ☐ Very regularly (90-100%)

Q9 Use of video-based online meetings?

- ☐ No
- ☐ Yes

Q10 Do you think that the same successes can be achieved by means of video-conference-based diagnostics versus therapy as with an analogue meeting?

Diagnostics

- ☐ worse
- ☐ same
- ☐ better

Therapy

- ☐ worse
- ☐ same
- ☐ better

Q11a Are there patient groups for whom you consider videoconferencing to be unsuitable or hardly suitable (for the diagnosis / therapy of acquired, neurogenic speech/communication disorders)?

- ☐ No
- ☐ Yes

Q11b If yes: which patient groups? [→ Free text entry]

Q12a Are there patient groups for whom you consider videoconferencing to be particularly applicable?

- ☐ No
- ☐ Yes

Q12b If yes: which patient groups? [→ Free text entry]

Section 3 Brain tumour patients

Q13 Do you generally also carry out diagnostics and/or therapy of speech/communication disorders in brain tumour patients? Please indicate their estimated proportion in your daily practice.

- ☐ Never
- ☐ Very rarely (< 1%)
- ☐ Rather rarely (1-14%)
- ☐ Sometimes (15-24%)
- ☐ Rather often (25% - 49%)
- ☐ Frequently (50% or more)

Q14a Do you think that the digital participation of (brain tumour) patients should be improved with regard to diagnostics and therapy of speech/ communication disorders?

- ☐ No
- ☐ Yes

Q14b If yes: How? [→ Free text entry]

Q15a Do you think that brain tumour patients are referred adequately often and sufficiently early for therapy of linguistic-communicative skills?

- ☐ neither nor
- ☐ not adequately often
- ☐ not sufficiently early
- ☐ yes

Q15b If not yes: Why? [→ Free text entry]
